# System-level FPGA validation of a trainable and robust multiplier-free spiking neural network

**DOI:** 10.1016/j.isci.2026.115985

**Published:** 2026-06-04

**Authors:** Qixuan Li, Lei Zhang

**Affiliations:** 1 Electronic Systems Engineering, University of Regina, Regina, SK, Canada

**Keywords:** FPGA implementation, FPGA-based learning architecture, multiplier-free design, noise-tolerant computing, Neural networks

## Abstract

The growing scale of artificial intelligence models has expanded their impact, yet efficient deployment on hardware remains challenging due to intensive matrix-based computation and energy demand. Spiking neural networks (SNNs), inspired by biological neural processing, offer an event-driven and energy-efficient alternative for neuromorphic computing. In this work, we present a system-level FPGA validation of a trainable, multiplier-free SNN that supports on-chip supervised learning under fixed-point constraints. By implementing a sensitive-noise threshold (SNT) mechanism and a limited remote supervised method (LReSuMe) on a Xilinx ZCU104 FPGA platform, the proposed design achieves high throughput, low power consumption, and preserved robustness to the noise. Weight visualization further indicates structured and interpretable learning behavior, supporting the reliability of hardware-based neuromorphic systems.

## Highlights

• BRAM-based training using SNT and LReSuMe in FPGA architecture
• Hardware-efficient learning without multipliers or DSP blocks
• Robust classification under noise with low hardware cost
• High-throughput and low-power training and inference in Vivado implementation

## Graphical abstract

**Figure:**
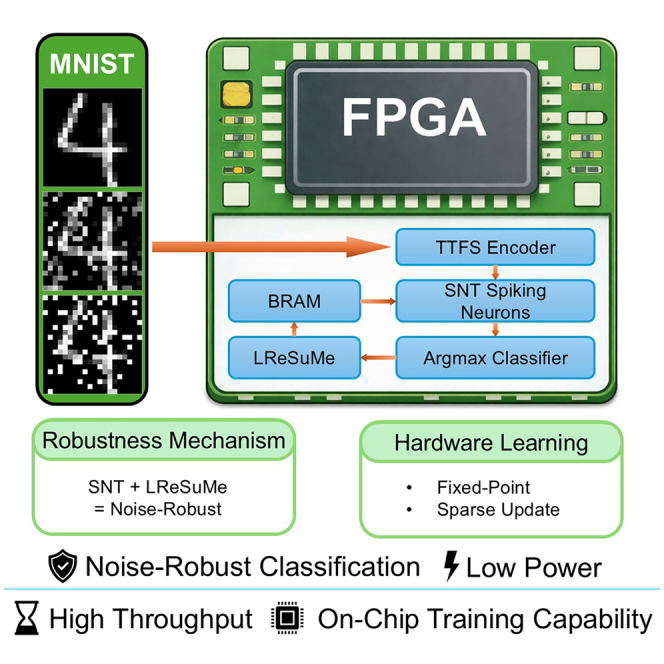

## Introduction

The rapid development of large-scale artificial intelligence (AI) models has led to remarkable progress in language, vision, and multimodal tasks. However, these models typically rely on billions of parameters and extensive matrix multiplications, resulting in slow training and excessive energy consumption.^1^^,^^2^ Hardware accelerators such as field-programmable gate arrays (FPGAs) and application-specific integrated circuits (ASICs) can parallelize feedforward computation,^3^ yet dense multiply-accumulate (MAC) workloads quickly exhaust limited on-chip resources.^4^ Moreover, the high computational and memory demands of gradient-based backpropagation (BP) make on-chip training impractical under strict power and resource constraints.^5^ These challenges motivate the exploration of learning paradigms that remain trainable while being compatible with hardware-oriented design flows. In contrast, the human brain processes multimodal sensory information in real time with a power budget of approximately 20 W, demonstrating both low latency and high energy efficiency.^6^ Among neuromorphic computing models, spiking neural networks (SNNs) emulate these characteristics most closely.

SNNs communicate through discrete temporal spikes rather than continuous activations, enabling event-driven computation, sparse activity, and improved energy efficiency.^7^^,^^8^ These properties naturally support noise tolerance and low-power operation, making SNNs promising candidates for edge intelligence. Despite these advantages, most existing FPGA-based SNN studies focus primarily on inference, while learning is commonly performed off-chip, approximated through artificial neural network (ANN)-to-SNN conversion,^9^ or restricted to unsupervised learning rules.^10^ As a result, the feasibility and numerical stability of on-chip supervised SNN training under realistic FPGA constraints remain insufficiently understood.

In our previous work,^11^ we proposed a simplified single-layer SNN that combines two key mechanisms: a sensitive noise-threshold (SNT), which dynamically adapts neuron firing thresholds to suppress noise-induced spikes, and a limited remote supervised method (LReSuMe), a lightweight, gradient-free learning rule that enables online training without BP.^12^ Software-based evaluations demonstrated that this approach can reduce training floating-point operations (FLOPs) by approximately 90% compared with a conventional ANN baseline, while maintaining competitive accuracy under severe noise corruption. These results suggest that the sparse update pattern and reduced arithmetic complexity of LReSuMe are particularly well suited to hardware-oriented implementations. However, software-level evaluation alone cannot capture the numerical precision limits, memory-access patterns, and control-flow constraints introduced by FPGA-oriented implementations. In particular, fixed-point arithmetic, limited fractional precision, and constrained weight ranges can fundamentally alter learning dynamics in ways that are not observable in floating-point models. Understanding these system-level effects is, therefore, essential for determining whether supervised learning rules remain stable and effective when deployed on hardware.

In this work, rather than proposing a new learning algorithm, we investigate the hardware realization of supervised SNN training by implementing and analyzing an LReSuMe-based learning framework under realistic FPGA constraints. We present a fully synthesizable, multiplier-free FPGA architecture that realizes an on-chip-compatible training datapath based on SNT and LReSuMe using fixed-point arithmetic. Implemented in very-high-speed integrated circuit hardware description language (VHDL) and validated through register-transfer level (RTL) and post-implementation simulations, the design enables quantitative evaluation of latency, resource utilization, power consumption, and numerical behavior under realistic FPGA constraints. The proposed system is evaluated using the MNIST dataset, with additional impulse noise injected into the input to assess robustness under fixed-point computation. Through this hardware-oriented validation flow, we identify precision-induced learning saturation, voltage- and weight-limited convergence, and strong sensitivity of online training dynamics to fractional bit-width, while demonstrating that the noise robustness of the LReSuMe-SNT framework is preserved after deployment on FPGA. Furthermore, the architecture clarifies why LReSuMe is particularly amenable to FPGA realization: its sparse, label-targeted updates significantly reduce memory-access frequency and control complexity compared with unsupervised learning rules such as spike-timing-dependent plasticity or gradient-based methods. The interpretable synaptic weight patterns observed after training further support the stability and meaningfulness of supervised learning under fixed-point constraints.

## Results

### Reference encoding strategies, SNN model, and learning rationale

In recent years, neuromorphic computing has evolved from neuron- and algorithm-level exploration toward system-level architecture design, driven by the demand for scalable, energy-efficient, and cognitively inspired intelligence.^13^ Contemporary neuromorphic systems increasingly emphasize large-scale integration, heterogeneous processing, and multi-level abstraction to support perception, decision-making, and learning within a unified framework. Such architectures aim to bridge biological plausibility and computational efficiency by leveraging event-driven processing and massive parallelism. However, as system complexity increases, learning mechanisms often become tightly coupled with high computational cost, complex control logic, or offline training pipelines.^14^ These constraints pose significant challenges for deploying neuromorphic intelligence on resource-limited hardware platforms such as FPGAs, particularly when on-chip training is required. As a result, lightweight and hardware-compatible neuromorphic systems that support supervised learning directly on-chip remain relatively underexplored.

Representative neuromorphic systems have explored diverse architectural and learning paradigms to achieve scalability and robustness. Large-scale digital neuromorphic architectures employing multi-compartment neurons have demonstrated enhanced biological fidelity and representational capacity,^15^ while cognition-oriented systems such as BiCoSS adopt multigranular designs to integrate perception, memory, and decision processes.^16^ Other studies investigate biologically plausible learning mechanisms, including dopamine-modulated neural networks,^17^ to emulate reward-driven adaptation, as well as neuromorphic frameworks that tightly couple visual perception with decision-making to achieve fault tolerance under noisy conditions.^18^ While these systems exhibit impressive functional richness and biological relevance, they typically rely on offline training, complex learning dynamics, or substantial computational resources. Such requirements significantly limit their applicability to lightweight, on-chip trainable FPGA systems, where resource efficiency, deterministic control flow, and numerical stability are critical design constraints.

Although SNNs are, by definition, more biologically inspired compared to conventional neural networks, their implementation on FPGAs can follow diverse design choices. To construct an SNN, one must first determine the encoding method and neuron model to be employed. In most existing FPGA-based SNN designs, the hardware is dedicated primarily to inference, while incorporating on-chip training remains less explored.^19^^,^^20^^,^^21^ When training is considered, the choice of encoding method and neuron model directly influences the selection of the corresponding training strategy. Furthermore, in terms of FPGA implementation metrics such as resource utilization and power consumption, different design approaches can lead to significantly different outcomes.

**Figure 1:**
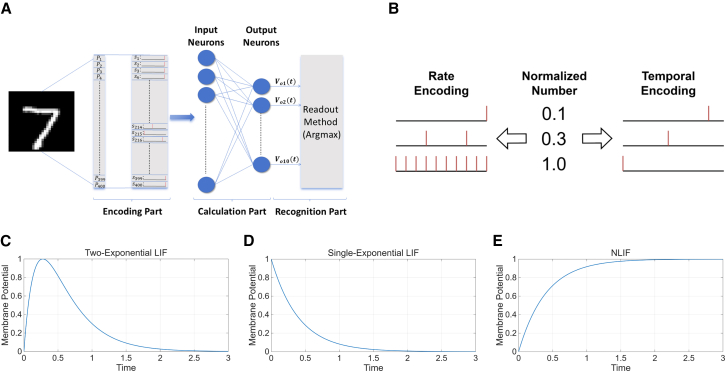
Background illustrations of the reference SNN model, encoding strategies, and neuron dynamics (A) Single-layer SNN architecture adopted in this work, originally proposed and evaluated in a software (MATLAB) environment in our previous study.^11^ The network consists of an input layer with 400 neurons and an output layer with 10 neurons, forming a fully connected 400 × 10 structure. (B) Temporal encoding encodes input magnitude into spike timing, whereas rate encoding uses spike frequency to represent input intensity. The figure illustrates how TTFS generates a single spike per pixel while rate encoding produces multiple spikes distributed over time. (C) Two-exponential integrate-and-fire neuron response, in which the membrane potential is shaped by both synaptic dynamics and membrane leakage, resulting in a bi-exponential rise-and-decay temporal profile. (D) Single-exponential integrate-and-fire neuron response, where only membrane leakage is considered, leading to a monotonic exponential decay of the membrane potential. (E) Non-leaky integrate-and-fire neuron response with exponential charging behavior induced by synaptic input dynamics.

To provide a concrete reference for subsequent discussions, Figure 1A illustrates the single-layer SNN architecture adopted in this study, which was originally proposed and evaluated in a software (MATLAB) environment in our previous work.^11^ The network consists of an input layer with 400 neurons and an output layer with 10 neurons, forming a fully connected 400 × 10 structure. The input neurons encode pixel-level features into spike representations, while the output neurons perform classification and synaptic weight updates during training.

#### Encoding method

The encoding method, which transforms input information into spike trains, is the first design choice to be determined when constructing an SNN. Various encoding methods have been proposed in the literature, such as temporal encoding,^11^^,^^22^ rate encoding,^23^^,^^24^ Ben’s spiker algorithm, step-forward spike encoding algorithm,^25^ and the pulsewidth modulation-based algorithm.^26^ Among them, temporal encoding and rate encoding are the two most widely used approaches for processing digital signals.

Figure 1B depicts a representative temporal encoding scheme known as time-to-first spike (TTFS). In TTFS, the magnitude of the input determines the precise timing of a single spike within the spike train. In contrast, rate encoding represents the input magnitude by modulating the frequency of multiple spikes distributed across the spike train. From an FPGA design perspective, where minimizing signal activity is often desirable, TTFS has the advantage of requiring fewer spikes. Equation 1, as presented in our previous work,^11^ shows how TTFS can linearly map image pixel values into spike timings.

(Equation 1) $$t_{n}=255-P_{i}$$

Here, *t*_*n*_ denotes the time of spike occurrence and *P*_*i*_ corresponds to the pixel intensity of the input image. However, a key limitation of TTFS lies in its strong dependence on a single spike representation, which makes it highly sensitive to noise. Therefore, when TTFS is adopted as the encoding method in FPGA-based SNN designs, the robustness of the model under noisy conditions must be carefully evaluated. The FPGA design introduced in this work leverages the SNT and the LReSuMe algorithm proposed in our previous work^11^ to overcome this drawback and enhance reliability under high-noise scenarios.

#### SNN neuron model

The neuron model determines how the membrane potential evolves within a spiking neuron and how the firing threshold is defined. Over the years, various neuron models have been proposed, ranging from the biophysically detailed Hodgkin-Huxley model^27^ to simplified models such as integrate-and-fire (IF),^24^ non-leaky integrate-and-fire (NLIF),^28^ leaky integrate-and-fire (LIF),^11^^,^^29^ and more recent extensions such as the parametric leaky integrate-and-fire^30^ and gated leaky integrate-and-fire.^31^ Among these, NLIF and LIF are the most fundamental and widely adopted due to their simplicity and scalability. Figures 1C–1E illustrate the differences between the two-exponential LIF (TELIF), the single-exponential LIF (SELIF), and the NLIF models. Correspondingly, the membrane potential dynamics of TELIF, SELIF, and NLIF can be expressed in closed-form, as shown in Equations 2, 3, and 4.

(Equation 2) $$V_{\mathrm{TELIF}}\left(t\right)=A\left(e^{-\left(t-t_{i}\right)/\tau_{m}}-e^{-\left(t-t_{i}\right)/\tau_{s}}\right)$$

(Equation 3) $$V_{\mathrm{SELIF}}\left(t\right)=A\left(e^{-\left(t-t_{i}\right)/\tau }\right)$$

(Equation 4) $$V_{\mathrm{NLIF}}\left(t\right)=A\left(1-e^{-\left(t-t_{i}\right)/\tau }\right)$$

The TELIF model describes the membrane potential as the difference of two exponential terms associated with the membrane and synaptic time constants, where the membrane time constant *τ*_*m*_ is typically larger than the synaptic time constant *τ*_*s*_, resulting in a bi-exponential temporal response that captures both the rising and decaying phases of neuronal activity. The SELIF model simplifies this formulation by retaining only a single exponential term to represent membrane leakage, resulting in a purely decaying temporal response following spike arrival. In contrast, the NLIF model removes membrane leakage and models the membrane potential as the cumulative integration of synaptic inputs, leading to an exponential charging behavior over time. These formulations provide different abstractions of membrane potential evolution while remaining analytically tractable for SNN modeling.

When implementing SNNs on FPGAs, the complexity of the chosen neuron model directly affects hardware implementation cost. For example, the TELIF model requires two exponential functions to describe membrane potential dynamics, whereas SELIF and NLIF can be represented using only a single exponential function. Although modern FPGA platforms provide IP cores such as coordinate rotation digital computer (CORDIC) for exponential calculations,^32^ these modules often require multiple clock cycles to compute each value, resulting in increased latency.

In discrete-time digital circuit implementations, the time difference *t* − *t*_*i*_ can be expressed as the fixed interval between consecutive time steps. Since both the time constant *τ* and the base of the exponential function are constants, the exponential decay term in the SELIF model can be precomputed and absorbed into a constant decay coefficient *D*. Based on this observation, our previous work^11^ proposed an optimized formulation for SELIF, shown in Equation 5, where the membrane potential update is implemented using a simple MAC operation, significantly reducing computational complexity.

(Equation 5) $$V_{j}\left(t_{n}\right)=V_{j}\left(t_{n-1}\right)*D+\overset{N}{\underset{i=1}{\sum }}s_{i}\left(t_{n}\right)*W_{i,j}$$

(Equation 6) $$s_{i}\left(t_{n}\right)=\left\{\begin{array}{ll} 1, & if\;a\;spike\;appear\;at\;t_{n}\\ 0, & otherwise. \end{array}\right)$$

In Equation 5, *W*_*ij*_ denotes the synaptic weight between the *i*th pre-synaptic neuron and the *j*th post-synaptic neuron, while *s*_*i*_(*t*_*n*_) represents the spike train generated by the encoding method (defined in Equation 6). *V*_*j*_(*t*_*n*_) represents the membrane potential in the current time step, and *V*_*j*_(*t*_*n*−1_) means the membrane potential for the previous time step. By constraining *D* to a fixed value, the hardware implementation becomes significantly more efficient without sacrificing model fidelity.

In most basic neuron models, the firing threshold is typically fixed at 1. However, our previous work^11^ introduced a variable threshold SNT. Unlike a fixed threshold that only determines spike occurrence, SNT evaluates the membrane potential over the threshold and accumulates the overshoot into a score. For the positive SNT (PSNT), early overshoots contribute positively to the score, while later overshoots introduce penalties. For the negative SNT (NSNT), early overshoots mean penalties, and later overshoots introduce awards. At the end of the simulation window, the output neuron with the highest accumulated score is selected as the predicted class. This scoring-based approach with the LReSuMe training method introduced in our previous work^11^ improves robustness under noisy input conditions.

#### Training method

From a hardware perspective, the distinction between supervised and unsupervised learning rules is not merely algorithmic, but directly impacts control complexity, memory-access frequency, and arithmetic cost in FPGA implementations. Unsupervised rules, such as spike-timing-dependent plasticity (STDP), require continuous tracking of pre- and post-synaptic spike timing relationships at the synapse level, with weight updates triggered by temporally correlated spike pairs.^33^ From a hardware perspective, maintaining such distributed and fine-grained temporal state across a large number of synapses leads to frequent state updates and nontrivial control logic, which can increase control overhead and memory activity in FPGA implementations. In contrast, gradient-based supervised methods rely heavily on multipliers and dense parameter updates,^34^ which are costly to implement on resource-constrained hardware. These considerations highlight the importance of learning rules whose update patterns and arithmetic structures align naturally with FPGA-oriented design principles.

Currently, many SNN inference tasks are deployed on FPGAs, whereas training remains difficult to implement in hardware. The main challenges stem from the non-differentiable and discrete nature of spikes. STDP,^35^ as a widely studied unsupervised learning rule, involves exponential functions in its formulation, which significantly complicates training when mapped to FPGA hardware. A previous study introduced an ANN-to-SNN conversion approach,^24^ where all pre-trained ANN parameters were directly transferred to an SNN for hardware inference. However, the training stage relied on BP, which requires extensive matrix multiplications—an operation highly resource-intensive on FPGAs. Similarly, Mostafa employed an NLIF neuron model and applied BP for training.^28^ Yet in such schemes, each trainable weight demands multiple multipliers, quickly exhausting FPGA resources.

As a continuation of our previous work,^11^ this paper adopts the LReSuMe approach to overcome the above limitations. LReSuMe extracts salient features from input images and directly encodes them into the synaptic weights. Throughout training, no gradient computation or differentiation of a loss function is required. Moreover, LReSuMe only updates the weights connected to the correct output neuron when the model misclassifies, thereby avoiding large-scale and frequent weight updates. This makes LReSuMe a promising candidate for FPGA-based on-chip training of SNNs. The detailed hardware mapping of this method will be presented in the next section.

In summary, prior studies on FPGA-based SNNs and neuromorphic systems have investigated a broad spectrum of encoding schemes, neuron models, and learning rules. However, when viewed from a hardware-oriented perspective, most existing approaches either prioritize inference acceleration or adopt learning mechanisms whose update patterns, arithmetic requirements, or control complexity are misaligned with resource-constrained FPGA platforms. In particular, the combined effects of spike encoding density, neuron dynamics, learning-rule-induced update sparsity, and numerical precision on on-chip supervised training behavior have not been systematically examined in existing FPGA-based SNN implementations. This gap motivates the present work, which focuses on the hardware realization and system-level behavior of an LReSuMe-based supervised SNN training framework, explicitly co-designed with TTFS encoding, simplified neuron dynamics, and fixed-point FPGA constraints.

### FPGA architecture and hardware implementation of the proposed SNN

#### SNN architecture on FPGA

**Figure 2:**
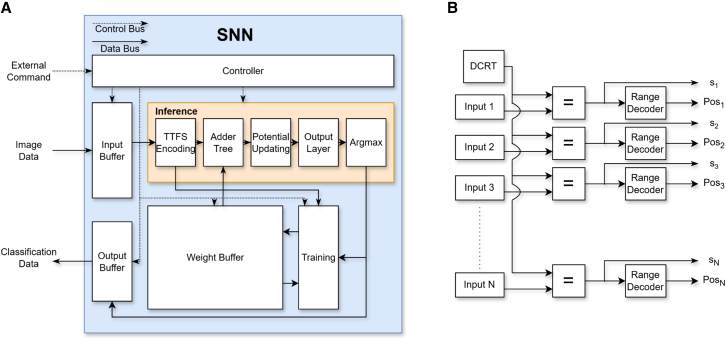
SNN architecture and TTFS encoding module implemented on FPGA (A) Overall FPGA-based implementation of the SNN system. An on-chip controller orchestrates the execution of inference and training submodules under external commands. Dashed arrows denote control signals, while solid arrows indicate data flow between functional modules. (B) Hardware structure of the TTFS encoder. Pixel inputs are compared with a global countdown counter to generate spike signals, and a range decoder extracts the relative spike timing to produce positional outputs *Pos*_*i*_.

To realize the proposed 400 × 10 SNN on FPGA, we design a modular architecture that maps each stage of the computation to dedicated hardware blocks. Figure 2A illustrates the flow of data and control signals across different modules. Within the overall SNN, operations such as repeated input encoding, weight accumulation in the adder tree, result storage address updates, weight retrieval from block RAM (BRAM), and weight update storage are all orchestrated by the controller. Once the controller receives an external command, the entire model begins execution.

The input data are 20 × 20 MNIST images obtained after edge cropping, and only one image is fed into the SNN module at a time. Since each pixel value ranges from 0 to 255, the input buffer is designed with an 8-bit input port, allowing the model to receive a complete image within 400 clock cycles. After the input buffer has stored an entire image, the data are transmitted in a fully parallel manner to the TTFS encoding module in the inference stage, where they are converted into spike trains. The encoded spikes are directly fed into the adder tree, which works together with the potential updating module and the output layer to perform SNN neuron computations.

To reduce hardware resource consumption, the SNN is implemented in a 400 × 1 configuration. Thus, by iterating the computation over 10 cycles, the design achieves an equivalent 400 × 10 functionality. During these iterations, the weight BRAM sequentially loads different groups of weights into the adder tree. After 10 iterations, the outputs are passed to the argmax module, which selects the maximum value to generate the final prediction for the input image. The prediction result is then delivered through the output buffer.

If the external command received by the controller includes a training request, the training module compares the predicted label with the correct label. If they match, no weight update is performed. If they differ, the training module retrieves the corresponding weights associated with the correct neuron from the weight BRAM and performs targeted updates.

#### TTFS encoding module

The primary function of the TTFS module can be expressed by Equation 1, while its hardware implementation is illustrated in Figure 2B. Figure 2B demonstrates how the 400 pixels from the input buffer are converted into spike trains *s*_*i*_, which can be described by Equation 6. Each input represents an 8-bit value and is connected to a dedicated comparator. The other terminal of each comparator is connected to a countdown counter initialized at 255 and decreasing to 0. Once the TTFS module receives an enable signal from the controller, the counter begins operation. When an input value matches the current counter value, the corresponding comparator generates a spike, which is then transmitted as *s*_*i*_ to the adder tree module. Furthermore, the spike is evaluated by a range decoder, which determines whether its generation time is greater than or equal to 250, between 1 and 249, or exactly 0, thereby producing the corresponding *Pos*_*i*_ signal. Equation 7 defines how the range decoder generates the binary value *Pos*_*i*_. The utilization of *Pos*_*i*_ will be further explained in the training design section.

(Equation 7) $$Pos_{i}=\left\{\begin{array}{ll} 11_{2}, & if\;255-t_{n}\geq 250\\ 10_{2}, & if\;249\geq 255-t_{n}\geq 1\\ 01_{2}, & if\;255-t_{n}=0\\ 00_{2}, & otherwise. \end{array}\right)$$

#### Neuron computation module

**Figure 3:**
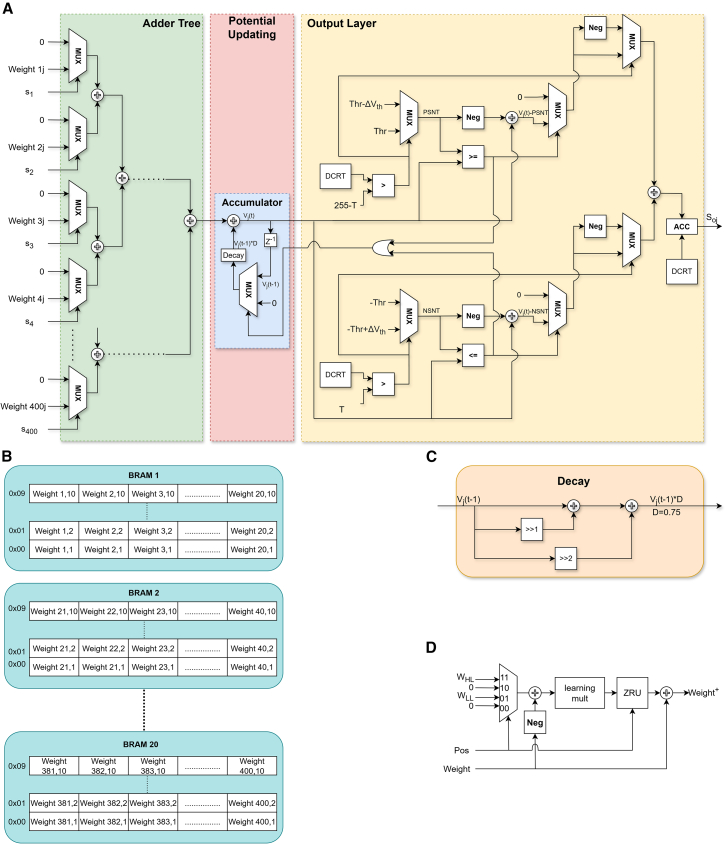
FPGA-based implementation of the neuron computation, weight storage, decay, and training modules (A) Neuron computation module implemented on FPGA, illustrating the datapath from TTFS-encoded spike trains to the final output score. The module consists of an adder tree for spike accumulation, a membrane potential updating unit, and an output layer that detects threshold crossings based on SNT and updates the output score accordingly. (B) Organization of 4,000 synaptic weights across 20 on-chip block RAMs (BRAMs). Each BRAM stores weights associated with a single output neuron, enabling parallel access to all corresponding synaptic weights during inference and training by fixing the neuron address. (C) Multiplier-free circuit for realizing a fixed decay coefficient *D* = 0.75 using a shift-and-add structure. Partial results generated by binary shifts are combined through addition to obtain the scaled output, representing a standard hardware-efficient implementation for constant multiplication in FPGA designs. (D) FPGA-based training module implementing fixed-point weight updates using an LReSuMe-based learning rule. The module includes a shift-and-add learning multiplication unit and a zero replacement unit (ZRU) to mitigate quantization-induced null updates.

The neuron computation module is primarily responsible for calculating the final output of each output neuron. The circuit design of this module is illustrated in Figure 3A. In this design, $\sum_{i=1}^{N}s_{i}\left(t_{n}\right)*W_{i,j}$ in Equation 5 is computed by the adder tree module, while the reset part of Equation 5 is handled by the potential updating module. The detection of membrane potential crossing the SNT, the corresponding computations, and the accumulation of neuron scores are all performed by the output layer module. After the computations for the ten output neurons are completed, the accumulated scores are passed to the argmax module, which compares the values to perform classification and outputs the predicted label to the output buffer.

In the following subsections, the adder tree, potential updating, output layer, and argmax modules are described in detail, highlighting their respective roles in implementing the neuron computation process.

##### Adder tree

In the first stage of the adder tree, 400 multiplexers (MUXs) are used to process the 400 spike trains received from the TTFS encoding module. Each spike train is connected to the selection port of its corresponding MUX. The two input ports of the MUX are connected to zero and to the weight retrieved from the weight BRAM, respectively. As expressed in Equation 6, each spike train contains only binary values of 0 or 1. When *s*_*i*_(*t*_*n*_) is 1, the MUX forwards the corresponding weight into the adder tree; otherwise, it outputs 0. Since the adder tree module needs to process all 400 spike trains, a total of nine addition layers are implemented.

The adder tree is employed primarily to achieve higher throughput. If sequential accumulation were used instead, the 400 spike trains generated by the TTFS encoding module would each require 256 clock cycles to complete, resulting in a significant increase in computation time per image. In contrast, the high degree of parallelism inherent in the adder tree effectively mitigates the inefficiency of sequential addition.

Although the highly parallel nature of the adder tree could generally lead to increased power consumption and computational load, the adoption of TTFS encoding ensures that each spike train contains only a single pulse. As a result, the combined use of the TTFS encoding module and the adder tree module avoids excessive overhead while maintaining efficient computation.

##### Potential updating

The potential updating module is responsible for updating the membrane potential within each spiking neuron. Its functionality is primarily realized by an accumulator. At each clock cycle, the value provided by the adder tree module is added to the membrane potential from the previous cycle, producing the updated potential for the current cycle. In conventional SNN designs, once the membrane potential crosses the threshold, it is forced to remain at zero for a certain duration, known as the refractory period. However, in the design illustrated in Figure 3A, the refractory period is set to zero. If this updated membrane potential reaches or exceeds any spike threshold (including either PSNT or NSNT), the output layer module asserts a signal to the selection port of the multiplexer inside the accumulator, forcing its output to zero. Otherwise, if no threshold is exceeded during the current cycle, the multiplexer outputs the current membrane potential. This output then passes through a delay element and a decay unit, before being added to the subsequent value from the adder tree module to generate the membrane potential of the next clock cycle. The entire update process can be formally described by Equations 8 and 9, and PSNT and NSNT will be introduced in detail in the following subsection.

(Equation 8) $$V_{j}\left(t_{n}\right)=U+\overset{N}{\underset{i=1}{\sum }}s_{i}\left(t_{n}\right)W_{i,j},$$

(Equation 9) $$U=\left\{\begin{array}{ll} V_{j}\left(t_{n-1}\right)*D, & if\;PSNT\geq V_{j}\left(t_{n-1}\right)\geq NSNT\\ 0, & otherwise. \end{array}\right)$$

According to Equation 9, when *V*_*j*_(*t*_*n*−1_) lies between NSNT and PSNT, the membrane potential is scaled by a decay coefficient *D* to obtain the updated potential. In the FPGA implementation, this constant multiplication is realized using a shift-and-add structure, as illustrated in Figure 3C.

Specifically, the decay module takes *V*_*j*_(*t*_*n*−1_) as input, where a right shift by one bit corresponds to division by two, and a right shift by two bits corresponds to division by four. By summing appropriately shifted versions of the input, fractional decay factors such as *V*_*j*_(*t*_*n*−1_) × 0.75 can be constructed without dedicated hardware multipliers. This multiplier-free realization follows a standard FPGA engineering practice for constant multiplication, which has been widely adopted in digital signal processing and arithmetic circuit design.^36^^,^^37^ By adopting this established shift-and-add approach in the proposed decay module, hardware resource utilization is significantly reduced compared to conventional multiplier-based implementations, while maintaining sufficient numerical accuracy for fixed-point SNN computations.

##### Output layer

The output layer module allows each output spiking neuron to monitor the magnitude of its membrane potential. As shown in Figure 3A, the upper part corresponds to computations in the positive potential domain, while the lower part handles those in the negative domain. Once the potential reaches or exceeds any SNT, the excess beyond the threshold is retained as the score for that time step, with its sign determined by the timing of the crossing. The score is then accumulated over time. When the countdown counter of the output neuron completes all 256 time steps, the final accumulated score is stored in a register. After the SNN module finishes processing for ten output neurons, these stored scores are passed to the argmax module for classification. The definitions of PSNT and NSNT for output spiking neurons are provided in Equations 10 and 11.

(Equation 10) $$PSNT=\left\{\begin{array}{ll} thr, & if\;255-t_{n}>255-T\\ thr-\Delta V_{th}, & otherwise. \end{array}\right)$$

(Equation 11) $$NSNT=\left\{ \begin{array}{c} -thr+{\Delta V}_{th},\;\;\;\;if\;255-t_{n}>T\\ -thr,\;\;\;\;\;\;\;\;\;\;\;otherwise. \end{array}\right.$$

In these equations, *thr* denotes the unmodified threshold, typically set to 1. Δ*V*_*th*_ represents the threshold difference used to adjust the threshold potential level, while *T* specifies the threshold partition that determines when the threshold changes. For the PSNT, the initial threshold is *thr*, which shifts to *thr* − Δ*V*_*th*_ once the countdown counter value falls below 255 − *T*. Similarly, for the NSNT, the initial threshold is −*thr* + Δ*V*_*th*_, which becomes −*thr* once the countdown counter value falls below *T*. These operations can be directly mapped onto hardware using comparators and multiplexers.

In the output layer module, the membrane potential is compared against the current PSNT and NSNT values, and the potential difference, Δ*V*_*PSNT*_(*t*_*n*_) and Δ*V*_*NSNT*_(*t*_*n*_), at a given time step is obtained by subtracting the corresponding threshold. A positive domain voltage score *V*_*Ps*_(*t*_*n*_) is generated only when *V*_*j*_(*t*_*n*_) exceeds the PSNT, and a negative domain voltage score *V*_*Ns*_(*t*_*n*_) is generated when *V*_*j*_(*t*_*n*_) falls below the NSNT. This functionality can be realized in hardware using comparators, adders, and multiplexers. Equations 12, 13, 14, and 15 can show the operation of these computation.

(Equation 12) $$\Delta V_{\mathrm{PSNT}}\left(t_{n}\right)=V_{j}\left(t_{n}\right)-PSNT$$

(Equation 13) $$\Delta V_{\mathrm{NSNT}}\left(t_{n}\right)=V_{j}\left(t_{n}\right)-NSNT$$

(Equation 14) $$V_{Ps}\left(t_{n}\right)=\left\{\begin{array}{ll} \Delta V_{\mathrm{PSNT}}\left(t_{n}\right), & if\;V_{j}\left(t_{n}\right)\geq PSNT\\ 0, & otherwise. \end{array}\right)$$

(Equation 15) $$V_{Ns}\left(t_{n}\right)=\left\{\begin{array}{ll} \Delta V_{\mathrm{NSNT}}\left(t_{n}\right), & if\;V_{j}\left(t_{n}\right)\leq NSNT\\ 0, & otherwise. \end{array}\right)$$

The timing of the threshold crossing then determines the sign of *V*_*Ps*_(*t*_*n*_) and *V*_*Ns*_(*t*_*n*_). Specifically, if the membrane potential exceeds the PSNT while the PSNT value is set to *thr*, the positive domain output score *V*_*Poj*_(*t*_*n*_) is a positive value; if it occurs when the PSNT has shifted to *thr* − Δ*V*_*th*_, the *V*_*Poj*_(*t*_*n*_) becomes a negative value. Conversely, if the membrane potential falls below the NSNT when its value is −*thr* + Δ*V*_*th*_, the negative domain output score *V*_*Noj*_(*t*_*n*_) is a negative value, whereas if it occurs when the NSNT is −*thr*, the *V*_*Noj*_(*t*_*n*_) is a positive value. Finally, the final score *S*_*oj*_ accumulated across all 256 time steps are stored in a register for subsequent use. This part of the design can be implemented using multiplexers, adders, and an accumulator, and its computation is summarized by Equations 16, 17, and 18.

(Equation 16) $$V_{Poj}\left(t_{n}\right)=\left\{ \begin{array}{cc} V_{Ps}\left(t_{n}\right), & if\;255-t_{n}>255-T\\ -V_{Ps}\left(t_{n}\right), & otherwise. \end{array}\right.$$

(Equation 17) $$V_{Noj}\left(t_{n}\right)=\left\{ \begin{array}{cc} V_{Ns}\left(t_{n}\right), & if\;255-t_{n}>T,\\ -V_{Ns}\left(t_{n}\right), & otherwise. \end{array}\right.$$

(Equation 18) $$S_{oj}=\overset{255}{\underset{n=0}{\sum }}V_{\mathrm{Poj}}\left(t_{n}\right)+V_{\mathrm{Noj}}\left(t_{n}\right)$$

It is worth noting that the countdown counter in the output layer module cannot be reused from the TTFS encoding module, since the signals pass through the adder tree and potential updating modules in between. These intermediate modules introduce clock-cycle delays through D flip-flops. Therefore, before the output layer module is activated, its internal countdown counter must be properly aligned with the clock cycle corresponding to its first input.

##### Argmax

After the adder tree, potential updating, and output layer modules have been executed ten times, *S*_*oj*_ of the ten output neurons are stored in registers. To achieve high throughput, the argmax module employs four layers of comparators to identify the maximum value: five comparators in the first layer, two in the second layer, one in the third layer, and one in the fourth layer. The argmax module then compares *S*_*oj*_ and selects the label associated with the maximum *S*_*oj*_. Unlike conventional argmax designs, this argmax module outputs a deliberately incorrect label when the maximum *S*_*oj*_ is equal to zero. A zero *S*_*oj*_ indicates that the membrane potential of the corresponding neuron has never exceeded any SNT during the entire sequence of time steps. If the controller in Figure 2A receives an instruction to train the SNN module, such incorrect labels will trigger the training module to update the weights.

#### Weight storage and management

Since the SNN module in this work is designed to perform a 400 × 10 computation, a total of 4,000 weights are stored in BRAM. To reduce output pressure, these weights are distributed across 20 BRAM blocks. Figure 3B illustrates the organization of weight storage within the BRAMs. In each BRAM, address 0 × 00 stores the 20 weights required by the first output neuron, address 0 × 01 stores the 20 weights required by the second output neuron, and so forth. When the controller issues a read signal along with an address, all 20 BRAMs simultaneously output the 400 weights corresponding to that address, which are then used by both the adder tree module and the training module. Among all components, the training module is the only one that modifies the weights. During the training stage, the controller of the SNN module sends a write signal and address to the BRAMs, enabling the training module to update the weights at the targeted addresses one by one.

#### Training design

In our previous work,^11^ LReSuMe demonstrated both efficiency and convenience for training a single-layer SNN. LReSuMe updates only the synaptic weights of the correct label neuron when misclassification happens, thereby significantly reducing the number of BRAM accesses required for weight storage as well as the time needed for weight updates in hardware implementations. The training procedure of LReSuMe for single-layer SNN can be summarized by Equations 19, 20, 21, and 22.

(Equation 19) $$W_{HL,j}=\frac{V_{\lim}}{400*\alpha_{j}/A_{j}}$$

(Equation 20) $$W_{LL,j}=\frac{-V_{\lim}}{400*\beta_{j}/A_{j}}$$

(Equation 21) $$\epsilon_{i,j}=\left\{\begin{array}{ll} W_{HL,j}-W_{i,j}, & if\;Pos_{i}=11_{2}\\ W_{LL,j}-W_{i,j}, & if\;Pos_{i}=01_{2}\\ 0-W_{i,j}, & otherwise. \end{array}\right)$$

(Equation 22) $$W_{i,j}^{+}=W_{i,j}+\eta *\epsilon_{i,j}$$

In Equation 19, *V*_lim_ denotes the voltage limitation, which is a parameter used to restrict the magnitude of the weights. *α*_*j*_ represents the number of pixels with intensity values greater than or equal to 250 in images belonging to class *j*, while *A*_*j*_ denotes the total number of pixels across all images in class *j*. The ratio *α*_*j*_/*A*_*j*_ can thus be interpreted as the percentage of pixels with intensity values greater than or equal to 250 within class *j*. Since the SNN module is designed for 20 × 20 MNIST images, the denominator in Equation 19 corresponds to the probability that a single image contains pixels with values greater than or equal to 250. Combined with *V*_lim_, this yields the high weight limitation for the *j*th output neuron, denoted as *W*_*HL*,*j*_.

Equation 20 defines the low weight limitation for the *j*th output neuron, denoted as *W*_*LL*,*j*_. Here, *β*_*j*_ represents the number of pixels with intensity values equal to zero in images of class *j*.

Equation 21 describes the calculation of the error term *ϵ*_*i*,*j*_. The formulation of *ϵ*_*i*,*j*_ varies depending on the value of *Pos*_*i*_ associated with the current weight *W*_*i*,*j*_. Once *ϵ*_*i*,*j*_ is obtained, it is multiplied by the learning rate *η* and then added to *W*_*i*,*j*_ to produce the updated weight $W_{i,j}^{+}$ in Equation 22.

Figure 3D illustrates the circuit design of the training module. Both *W*_*HL*,*j*_ and *W*_*LL*,*j*_ are precomputed constant values, and therefore, in hardware design, they are stored within the circuit and retrieved when needed. The design principle of the learning multiplication module follows the same shift-and-add approach introduced in the decay module of Figure 3C. In general, the circuit structure in Figure 3D is closely aligned with the formulations in Equations 19, 20, 21, and 22. The only exception is the inclusion of a zero replacement unit (ZRU). The entire circuit operates in fixed-point arithmetic with int integer bits and *frac* fractional bits (i.e., Qint.frac). When the number of fractional bits is insufficient, the output of the learning multiplication module often becomes zero (since *η*∗*ϵ*_*i*,*j*_ < 2^−*frac*^), resulting in no weight update. The ZRU addresses this issue by forcing the least significant bit (LSB) of the weight update to 1 when the output of the learning multiplication module is zero, thereby assigning the update the minimum nonzero magnitude. Equation 23 describes the role of the ZRU in this process, and *Q*(.) is the fixed-point quantization.

(Equation 23) $$\hat{\eta \varepsilon_{i,j}}=sign\left(\eta \varepsilon_{i,j}\right)\cdot \max\left(\left|Q\left(\eta \varepsilon_{i,j}\right)\right|,2^{-frac}\right)$$

When the SNN module is required to perform training, and the circuit produces an incorrect prediction for a given image, the training module updates the weights associated with the neuron corresponding to the correct label one by one. Unlike the fully parallel design adopted in the TTFS encoding module, this design choice stems from the fact that weights typically require a larger bit-width representation. Implementing a fully parallel architecture for training would significantly increase both resource usage and power consumption. Although the non-parallel approach requires the training module to operate for 400 clock cycles to complete one round of weight updates, the LReSuMe algorithm does not mandate weight updates after every single image prediction. As a result, this design does not substantially affect the overall throughput of the SNN module.

### Training, robustness, implementation, and interpretability results

#### Noise modeling and dataset preparation

**Figure 4:**
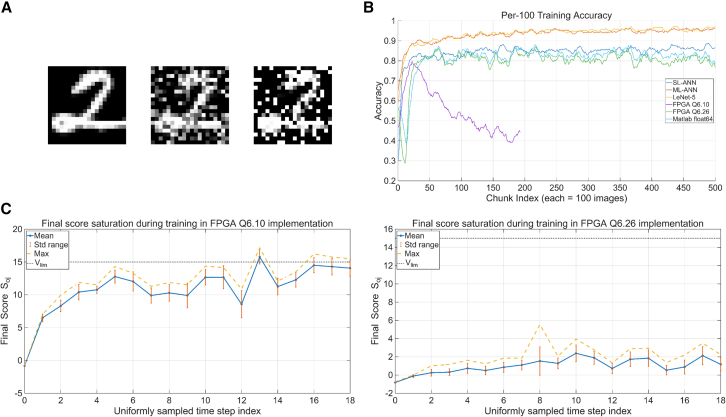
Noise modeling, training accuracy comparison, and fixed-point saturation behavior (A) Illustration of impulse noise modeling in the MNIST dataset. From left to right: original image, image corrupted with random impulse noise (random positions and random values), and image corrupted with impulse noise (random positions with pixel values replaced by either 0 or 255). (B) Training accuracy comparison among six different network configurations and quantization settings on the clean MNIST training dataset. Accuracy is reported per 100-image chunk. (C) Evolution of final output scores under different fixed-point formats during training. Results are shown for FPGA Q6.10 (left) and Q6.26 (right) implementations. The *x* axis denotes the uniformly sampled time step index selected from the first 180 training chunks of the hardware simulation, and the *y* axis represents the final output score $S_{o_{j}}$. At each sampled time step, the maximum, mean, and standard deviation of $S_{o_{j}}$ across all output neurons are computed and visualized.

The SNN module in this study utilizes a cropped version of the MNIST dataset, where each image is resized to 20 × 20 pixels after removing the blank borders. A total of 50,000 training images are used to train the SNN module and evaluate its learning accuracy. To reduce computational overhead, only a randomly selected subset of the 10,000 test images is used to assess and compare the model’s robustness to noise. In practice, evaluating the model on a subset of the test set is sufficient to reflect its overall accuracy and noise tolerance. Two types of noise are introduced to the images: random impulse noise, in which pixels at non-repeated random positions are replaced with random intensity values, and impulse noise, in which pixels at non-repeated random positions are replaced with either 0 or 255 to emulate salt-and-pepper distortion. Figure 4A shows an example of an original MNIST image, the same image corrupted with 150 random impulse noise points, and the same image corrupted with 150 impulse noise points.

#### Baseline configurations

The proposed model was implemented in VHDL and validated through RTL-level and post-implementation simulations using Xilinx Vivado 2025.1, targeting a Zynq UltraScale+ ZCU104 evaluation board (xczu7ev-ffvc1156-2-e). All hardware simulations were conducted with a system clock frequency of 100 MHz. The neuron model adopted a decay coefficient *D* of 0.75, and within the SNT module, the threshold *thr* was set to 1, the threshold difference Δ*V*_*th*_ to 0.6, and the threshold partition *T* to 20. In the LReSuMe-based training module, the voltage limitation *V*_lim_ was set to 15, and the learning rate *η* was configured to 0.00390625 (i.e., 2^−8^). To evaluate the numerical precision of the proposed design, two fixed-point configurations, Q6.26 and Q6.10, were used during both training and testing phases. These configurations were used to assess the trade-off between computational accuracy and hardware efficiency at different quantization levels.

To comprehensively evaluate the proposed model, three ANN architectures were implemented on software for comparison: a single-layer ANN (SL-ANN), a multi-layer ANN (ML-ANN), and a LeNet-5 convolutional network. The architecture of the SL- ANN is 400 × 10; the ML-ANN is a deeper network with 400, 256, 128, and 10 neurons; and the LeNet-5 model, consisting of two convolutional layers, two subsampling layers, and two fully connected layers, is used as a representative convolutional neural network to benchmark against the proposed model.^38^ To ensure consistency with the training strategy used in the proposed SNN module, all the aforementioned software-based networks were trained using online learning, in which the model parameters are updated after processing each image. All training and evaluation processes for these networks were conducted in a Python-based environment using the PyTorch^39^ and TensorFlow^40^ frameworks, with all computations performed in single-precision (32-bit) floating-point arithmetic.

In addition to the ANN baselines, a software-based SNN model corresponding to our previous work^11^ is also included for comparison. This software SNN adopts the same network architecture, encoding method, neuron model, and learning rule as the proposed FPGA implementation. All hyperparameters are configured identically to those used in the hardware-oriented design. The software SNN is implemented in MATLAB using 64-bit floating-point arithmetic and trained under the same online learning scheme. This configuration enables a controlled comparison between software-level and FPGA-oriented realizations of the same SNN model, allowing the impact of fixed-point quantization and hardware constraints on training behavior to be isolated and analyzed.

#### Training accuracy and fixed-point saturation behavior

Figure 4B compares the training accuracy of three software-implemented ANNs, a software-based SNN model, and the proposed SNN module implemented on FPGA hardware. All models were trained for a single epoch, with every 100 images grouped into one chunk to calculate chunk-wise accuracy. The resulting accuracy curves were then smoothed using a moving window of 10 for clarity. As expected, both the ML-ANN and LeNet-5 achieved high training accuracy due to their deeper architectures. In contrast, the SL-ANN and the proposed SNN design with a Q6.26 fixed-point representation exhibited comparable accuracy levels.

In addition to the ANN baselines, Figure 4B also includes the training curve of a software-based SNN model from our previous work.^11^ As shown in the figure, the software SNN achieved slightly higher training accuracy than the FPGA-based Q6.26 implementation, while exhibiting very similar convergence behavior. This observation indicates that the small performance gap mainly originates from numerical implementation differences, rather than from the learning algorithm itself.

Comparing the two FPGA configurations, Q6.26 and Q6.10, it can be observed that the accuracy of the Q6.10 design begins to drop sharply after approaching approximately 80% accuracy. This degradation is closely related to the limited fractional precision available for weight updates in the Q6.10 format. Insufficient fractional bits increase the likelihood of zero-valued updates in the learning multiplication module. Although the ZRU shown in Figure 3D alleviates this issue during the early stages of training, prolonged training gradually drives the synaptic weights toward their predefined limits, $W_{HL_{j}}$ and $W_{LL_{j}}$, as defined in Equations 19 and 20.

The effect of these weight limitations is further illustrated in Figure 4C. Since the weight limits are directly determined by the voltage limitation *V*_lim_, synaptic weights approaching their limits result in output neuron scores $S_{o_{j}}$ that increasingly concentrate near *V*_lim_. In the Q6.10 implementation, Figure 4C shows that both the mean and maximum output scores rapidly approach *V*_lim_ as training progresses, indicating a saturation effect induced by fixed-point constraints. Once the output scores are saturated, the discriminative capability of the output neurons is significantly reduced, leading to distorted learning behavior and a rapid decline in training accuracy after approximately 25 training chunks, as observed in Figure 4B. For the same reason, the Q6.10 configuration fails to complete training over the entire dataset, as excessively high output scores trigger premature termination of the training process.

In contrast, the Q6.26 implementation maintains output neuron scores well below *V*_lim_ throughout training, as shown in Figure 4C. This wider dynamic range allows the synaptic weights to evolve more smoothly without prematurely reaching their limits, thereby preserving stable learning dynamics and sustained training accuracy. As for the Q6.26 training curve in Figure 4B, an initial drop in accuracy can be observed before it quickly rises to a level comparable to that of both the SL-ANN and the software-based SNN. This phenomenon results from the initial weights being small positive values close to zero, which causes the output neurons to generate negative scores in the early training phase. As training progresses, these neuron outputs transition from negative to zero and eventually to positive values. During the brief valley period observed in the early stage of the Q6.26 training curve, most output neuron scores remain close to zero, which is consistent with this gradual dynamic adjustment process.

#### Hardware inference simulation verification

During the verification process, the proposed SNN module was not trained directly within the Vivado environment. Instead, the network weights were pre-trained in MATLAB and subsequently imported into the Vivado simulator for hardware-level verification. The trained weights were stored in the on-chip BRAM of the SNN module and utilized during inference simulation. This software-trained, hardware-simulated flow enables precise evaluation of the SNN’s behavior under fixed-point arithmetic while significantly reducing the computational overhead of on-board training.

##### Random impulse noise evaluation

**Figure 5:**
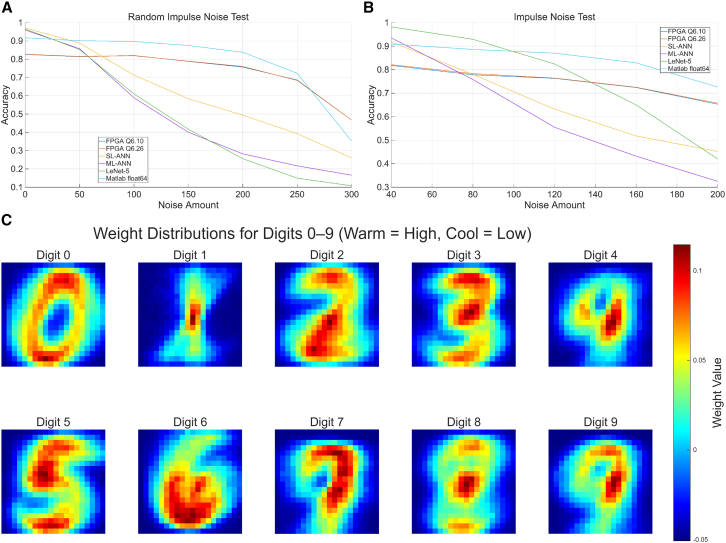
Noise robustness evaluation and weight interpretability of the proposed SNN (A) Classification accuracy under different levels of random impulse noise, where a number of pixels at random locations are replaced with random intensity values. (B) Classification accuracy under different levels of impulse noise, where pixels at random locations are replaced with binary values (0 or 255), emulating salt-and-pepper distortion. (C) Visualization of learned synaptic weights for output neurons corresponding to digits 0–9. The trained weight vectors are reshaped into 20 × 20 maps to reflect the spatial structure of the input images. Warm and cool colors indicate high and low synaptic weights, respectively, highlighting the interpretability and class-specific feature learning behavior of the proposed algorithm.

Figure 5A illustrates the test accuracy of different network architectures under varying levels of random impulse noise. Each data point represents the model’s performance when a given number of randomly selected pixels in the input image were replaced by random grayscale values ranging from 0 to 255. As the noise level increases, all models experience a gradual decline in accuracy; however, the proposed FPGA-based SNN module, particularly with the Q6.26 fixed-point representation, exhibits consistently higher robustness compared with the software-implemented ANNs.

The SNN module with Q6.26 maintains stable performance even when the noise level reaches 200 randomly corrupted pixels, whereas the accuracy of SL-ANN, ML-ANN, and LeNet-5 drops sharply beyond this point. This resilience is mainly attributed to the temporal and event-driven computing nature of the SNN and LReSuMe. It is also noteworthy that the difference in accuracy between the Q6.26 and Q6.10 configurations is minimal, indicating that even with fewer fractional bits, the inference-only version of the proposed design can still achieve a high level of noise robustness. This finding suggests that, in practical deployment scenarios where only inference is required, adopting a lower-precision fixed-point format can effectively reduce hardware cost and power consumption without significantly compromising accuracy.

In addition to the FPGA-based implementations, Figure 5A also includes the performance of a software-based SNN implemented in MATLAB.^11^ Under noise-free or low-noise conditions, the MATLAB-based SNN achieves slightly higher accuracy than its FPGA counterparts, which can be attributed to its use of high-precision floating-point arithmetic. However, as the noise level increases, the accuracy of the floating-point SNN degrades more rapidly than that of the FPGA-based SNNs. In particular, the fixed-point implementations maintain a more gradual performance decline under severe noise corruption, indicating improved robustness at high noise levels.

##### Impulse noise evaluation

Figure 5B illustrates model performance under impulse noise, in which randomly selected pixels are replaced by either 0 or 255, producing severe black-and-white corruption. Compared with random impulse noise, this type of noise introduces stronger local discontinuities and disrupts the structural continuity of the input images. As the noise level increases, all models exhibit a monotonic decrease in accuracy; however, the FPGA-based SNN module demonstrates a noticeably slower degradation rate and maintains more stable performance under heavy noise conditions.

For the FPGA implementations, the accuracy difference between the Q6.26 and Q6.10 configurations remains within 1% across all tested noise levels. The proposed SNN surpasses both the SL-ANN and the ML-ANN once the number of impulse noise pixels exceeds 80, and further outperforms the LeNet-5 model when the noise level is greater than 140. These results indicate that the proposed design exhibits superior robustness under extreme black-and-white corruption.

In addition, a trend consistent with Figure 5A is observed when comparing the MATLAB-based SNN with the FPGA implementations. At low impulse noise levels, the MATLAB-based SNN achieves slightly higher prediction accuracy. However, its accuracy curve shows a steeper decline as the noise amount increases, whereas the fixed-point FPGA-based SNNs degrade more gradually. Consequently, when the number of impulse noise pixels reaches 200, the prediction accuracy of the MATLAB-based SNN becomes comparable to that of the FPGA-based implementations. This behavior suggests that the fixed-point SNN maintains a more stable response under severe impulse noise, even when the absolute accuracy difference is small.

#### FPGA implementation and performance evaluation

Table 1 presents a comprehensive comparison of FPGA-based SNN implementations in terms of network scale, resource utilization, performance, and energy efficiency. The two proposed configurations are implemented on the same Zynq ZCU104 platform using identical network structures (400–10) and the same LReSuMe-based on-chip training framework, differing only in fixed-point precision (Q6.26 and Q6.10). This enables a direct evaluation of numerical precision trade-offs under otherwise identical hardware conditions. For the proposed implementations, hardware resource utilization, power consumption, and throughput are obtained from post-implementation reports generated by Xilinx Vivado. The energy consumption per image is computed by multiplying the measured power by the corresponding training or inference runtime.

**Table 1:** Comparison of FPGA-based SNN implementations: Resource utilization and performance

	Proposed	Proposed	FPGA design (Euler)^41^	FPGA design (RK3)^41^	FPGA design^42^	FPGA design^24^
FPGA device	Zynq ZCU104	Zynq ZCU104	Virtex-7 VC709	Virtex-7 VC709	Artix-7	xcku035-sfva784-2-e
Network configuration	400–10	400–10	784-100(Exc)-100(Inh)	784-100(Exc)-100(Inh)	784–400	401∗10
Dataset	MNIST	MNIST	MNIST	MNIST	MNIST	MNIST
Test accuracy	82.9%	81.6%	86.5%	86.5%	74.0%	90.4%
Data format	fixed Q6.26	fixed Q6.10	fixed Q16.9	fixed Q16.9	fixed Q13.3	fixed Q7.23
Neuron model	LIF	LIF	LIF	LIF	LIF	IF
Training method	LReSuMe	LReSuMe	STDP	STDP	STDP	BP (ANN2SNN)
LUTs	24,907	12,306	45,531	88,053	53,200	73,677
FFs	24,892	14,054	50,614	91,281	106,400	32,853
DSPs	0	0	–	–	–	10
BRAMs	180	90	289	289	140	0
Power (W)	0.366 + 0.593	0.214 + 0.593	–	–	–	1.131
Training runtime (s/img)	3.478 × 10^−5^	3.478 × 10^−5^	0.053	0.029	–	–
Inference runtime (s/img)	3.066 × 10^−5^	3.066 × 10^−5^	0.035	0.024	2.15 × 10^−4^	1.0 × 10^−4^
Training energy (mJ/img)	3.34 × 10^−2^	2.81 × 10^−2^	7.37	6.78	–	–
Inference energy (mJ/img)	2.94 × 10^−2^	2.477 × 10^−2^	4.45	1.74	13	0.1131
On-chip training capability	yes	yes	yes	yes	no	no

Both configurations achieve extremely low computational latency, with training and inference runtimes in the order of 10^−5^s per image, corresponding to a processing throughput exceeding 30,000 frames/s; it is owing to the label-triggered weight update mechanism of LReSuMe, resulting in significantly reduced hardware cost and training energy consumption. Compared with STDP-based FPGA implementations using Euler or RK3 solvers,^41^ which adopt larger multi-population network architectures to support unsupervised learning, the proposed designs achieve over two orders of magnitude lower training energy.

While some prior works^24^^,^^42^ focus exclusively on inference and rely on off-chip training, the proposed architecture supports fully on-chip supervised learning with substantially lower energy per inference. Although the achieved classification accuracy is slightly lower than that of BP-trained or large-scale STDP-based designs, the proposed implementations offer a more favorable balance among accuracy, energy efficiency, and hardware resource usage. Notably, reducing the fixed-point precision from Q6.26 to Q6.10 further decreases dynamic power and BRAM usage with negligible impact on accuracy, highlighting the robustness of the proposed architecture to aggressive quantization.

Comparing with the FPGA design with IF model,^24^ which adopts a network scale comparable to the proposed 400–10 SNN and achieves higher classification accuracy, the key distinction lies in training capability and system-level efficiency. The design proposed by Zhang and Zhang^24^ does not support on-chip training and relies on off-chip BP-based learning. Even under inference-only operation, it consumes more hardware resources and exhibits higher power consumption and lower throughput than the proposed implementations, as summarized in Table 1.

#### Weight interpretability analysis

By extracting the trained synaptic weights from the proposed SNN model and rearranging them according to their spatial order into a 20 × 20 matrix, the weight distribution of each neuron can be visualized, as shown in Figure 5C. In the figure, pixels with warmer colors represent weights closer to *W*_*HL*_, whereas cooler colors correspond to weights approaching *W*_*LL*_. It can be observed that, after LReSuMe-based training, the spatial weight patterns of individual neurons resemble the handwritten digits they are responsible for recognizing. This visualization demonstrates that the proposed training scheme enables the SNN to form class-specific receptive fields, making the network’s behavior more interpretable. This interpretability facilitates an intuitive understanding of how different neurons specialize in distinct digit categories. It provides a useful diagnostic tool for analyzing learning dynamics, feature selectivity, and potential redundancy within the spiking network.

## Discussion

This work extends our previous algorithm-level study on SNT-LReSuMe-based SNNs by examining their behavior and feasibility under realistic FPGA constraints. While the core learning rule and neuron dynamics have been reported previously,^11^ the present study shifts the focus from algorithmic performance to hardware-oriented system validation, addressing how numerical precision, architectural constraints, and on-chip training mechanisms jointly influence learning behavior. By implementing the complete training and inference pipeline on FPGA, including TTFS encoding, LIF neurons, SNT, and LReSuMe, this work provides a concrete assessment of the trade-offs between learning accuracy, hardware efficiency, and robustness that cannot be captured through software simulation alone.

To better understand the observed robustness of the proposed framework, we analyze its underlying mechanism. The robustness mainly originates from the combined effect of SNT and LReSuMe, rather than from TTFS encoding alone. TTFS is inherently sensitive to input perturbations, since each pixel is represented by a single spike whose timing can be directly affected by noise. The SNT mechanism mitigates this issue through its two-level PSNT and NSNT design, which partitions spike responses across different temporal regions. In particular, it emphasizes responses at early and late time steps while suppressing intermediate responses that are more susceptible to noise-induced fluctuations. This temporal selectivity enables the network to focus on more reliable spike events and capture class-specific features more effectively. Meanwhile, LReSuMe reinforces these discriminative patterns by embedding salient image features into the synaptic weights through sparse supervised updates. The resulting weight structures highlight spatial patterns associated with different digit classes, allowing the network to maintain reliable inference even under impulse noise.

A key finding of this study is that the robustness of the SNT-LReSuMe framework to impulse noise is largely preserved after deployment on FPGA, despite the use of fixed-point arithmetic. While prior results demonstrated noise tolerance at the algorithmic level, the present work confirms that this property is not an artifact of floating-point computation. In fact, under severe impulse noise, the fixed-point FPGA implementation exhibits a more gradual degradation trend compared with floating-point software models. This observation highlights an important system-level insight—hardware-induced numerical constraints do not necessarily weaken robustness and may, in some regimes, stabilize learning and inference behavior.

The comparison between LReSuMe and commonly used unsupervised learning rules such as STDP further clarifies the suitability of supervised learning for on-chip FPGA implementation. Although STDP updates are locally defined at the synapse level, practical FPGA realizations often require continuous monitoring of spike timing relationships across large neuron populations, leading to high memory access frequency and control complexity. Moreover, STDP-based designs frequently rely on expanded output layers with excitatory and inhibitory neuron populations to achieve stable competitive learning, which substantially increases network scale and hardware cost. In contrast, the supervised and label-triggered nature of LReSuMe confines weight updates to a single output neuron and only when misclassification occurs. This selective update pattern significantly reduces training activity, memory access, and power consumption, making LReSuMe more compatible with resource-constrained FPGA systems.

Another important observation arises from the fixed-point training behavior. Experimental results show that insufficient fractional precision leads to premature weight saturation and degraded learning performance even when mitigation mechanisms such as the ZRU are applied. As demonstrated by the evolution of output neuron scores, limited bit-width causes synaptic weights to approach their predefined bounds, which in turn compresses the dynamic range of output scores and distorts class discrimination. This behavior explains the abrupt accuracy degradation observed in low-precision configurations and underscores that bit-width selection is not merely an implementation detail but a critical design parameter for on-chip training stability.

In addition to performance metrics, the interpretability of the learned synaptic weights provides further evidence of stable and meaningful learning under fixed-point constraints. As shown in Figure 5C, output neurons trained with the LReSuMe rule develop structured, class-specific spatial patterns that resemble the underlying digit shapes. These patterns are preserved in the FPGA-trained model, indicating that the observed learning behavior is not a numerical artifact caused by fixed-point quantization. Instead, the weight structure reflects consistent feature encoding, reinforcing the reliability of the proposed hardware-oriented training framework.

From a system-design perspective, these findings position the proposed architecture as a reference for algorithm-hardware co-design rather than a standalone algorithmic contribution. The multiplier-free datapath, sparse supervised updates, and interpretable weight evolution together illustrate how learning rules can be selected and adapted to align with FPGA constraints. While the present implementation targets a relatively small single-layer SNN, the insights gained regarding numerical precision, update sparsity, and robustness provide practical guidance for scaling toward deeper or hybrid architectures in future work.

### Limitations of the study

Despite its advantages, the proposed design has several inherent limitations. First, the current implementation is restricted to a single-layer SNN with a network scale of 400 × 10, which limits its representational capacity compared with deep ANN or multi-layer SNN models. This architectural constraint partly explains the lower accuracy observed under noise-free conditions. Second, the LReSuMe learning rule cannot propagate error information across multiple layers, which restricts the proposed on-chip training framework to shallow network structures and limits its ability to model complex decision boundaries. This algorithmic limitation directly impacts scalability and prevents straightforward extension to deep SNN architectures. Third, the use of supervised learning requires access to labeled data during training, which limits applicability in fully autonomous edge scenarios. In practice, the proposed design is more suitable for controlled environments, calibration phases, or semi-supervised settings where supervision is intermittently available. Finally, the FPGA architecture in this work is optimized for fast inference and efficient on-chip training through fixed functional modules, such as the decay and training units. While this design choice improves throughput and reduces control overhead, it also limits flexibility in modifying internal parameters at runtime. As a result, adapting the proposed FPGA implementation to different neuron dynamics or learning configurations may require re-synthesis or reconfiguration of the hardware.

## Resource availability

### Lead contact

Requests for further information and resources should be directed to and will be fulfilled by the lead contact, Qixuan Li (li787@uregina.ca).

### Materials availability

No new physical or biological materials were generated in this work.

### Data and code availability

• The MNIST dataset used in this study is publicly available from Kaggle: https://www.kaggle.com/datasets/hojjatk/mnist-dataset.
• The N-MNIST dataset has been deposited in IEEE DataPort and is publicly available at https://doi.org/10.21227/0cbz-n616.
• The VHDL source code used in this study has been deposited in Zenodo and is publicly available at https://doi.org/10.5281/zenodo.17470023.
• Software tools used for implementation and evaluation, including Vivado, MATLAB, and Python, are listed in the key resources table.
• Any additional information required to reanalyze the data reported in this paper is available from the lead contact upon request.

## Acknowledgments

This research received no specific grants from any funding agency in the public, commercial, or not-for-profit sectors.

## Author contributions

Conceptualization, Q.L. and L.Z.; methodology, Q.L.; writing – original draft, Q.L.; writing – review and editing, Q.L. and L.Z.; and supervision, L.Z.

## Declaration of interests

The authors declare no competing interests.

## STAR★Methods

### Key resources table

**Table:** 

REAGENT or RESOURCE	SOURCE	IDENTIFIER
**Deposited data**		

MNIST	Kaggle	Kaggle MNIST: https://www.kaggle.com/datasets/hojjatk/mnist-dataset
N-MNIST	IEEE DataPort	N-MNIST: https://doi.org/10.21227/0cbz-n616

**Software and algorithms**		

Modified TTFS	Li and Zhang^11^	N/A
SNT	Li and Zhang^11^	N/A
LReSuMe	Li and Zhang^11^	N/A
Argmax classifier	Standard method	N/A
Vivado 2025.1	Xilinx	AMD Vivado: https://www.amd.com/en/products/software/adaptive-socs-and-fpgas/vivado.html
MATLAB 2025a	MathWorks	https://www.mathworks.com
Python 3.10.11	Python	https://www.python.org
VHDL	IEEE	IEEE Std 1076-2008
VHDL code	Zenodo	https://doi.org/10.5281/zenodo.17470023

### Method details

#### Datasets and preprocessing

Experiments were conducted using the MNIST handwritten digit dataset and the N-MNIST dataset generated in this study. The MNIST dataset was obtained from a publicly available repository, and the N-MNIST dataset was generated and released through IEEE DataPort. For MNIST, each input image was center-cropped from 28 × 28 pixels to 20 × 20 pixels by removing the outer borders. The resulting 400 pixel intensity values were directly provided to the FPGA-targeted architecture implemented in simulation. No additional feature extraction or normalization was applied.

#### FPGA-based TTFS encoding

TTFS encoding was implemented within the FPGA architecture. Each pixel intensity was mapped to a spike timing within a predefined temporal window. Higher pixel values produced earlier spikes, while lower values generated later spikes. Each input pixel generated at most one spike event during the encoding window. The TTFS encoding used in this work is a modified version of the conventional TTFS scheme, adapted for hardware implementation. The modification enables efficient mapping to FPGA logic and reduces control complexity during spike generation.

#### SNN architecture

The proposed architecture employs a single-layer spiking neural network consisting of 400 input neurons and 10 output neurons. Each input neuron corresponds to one pixel of the 20 × 20 input image.

During inference, input spikes generated by TTFS encoding control the selection of synaptic weights, which are conditionally accumulated at the output neurons. When a spike is present, the corresponding weight from BRAM is added to the membrane potential; otherwise, no update is performed. The neuron membrane potential evolves across the temporal window, and classification is performed using an argmax operation over output neuron responses.

#### SNT mechanism

To improve robustness and stabilize training under noisy inputs, the SNT mechanism was applied to the output neurons. The SNT consists of PSNT and NSNT, each defined with two threshold levels. These thresholds are used to detect the degree to which the membrane potential exceeds the specified threshold regions. This design increases the sensitivity of the network to temporally inconsistent or noise-induced spikes during training.

#### LReSuMe supervised learning

Training was performed using the LReSuMe. During training, synaptic weights were updated only when misclassification occurred. The update rule modifies the weights associated with the target output neuron according to the temporal region of input spikes. Spike events are partitioned into predefined time intervals, and weight updates are applied based on the region in which each spike occurs. This sparse update strategy reduces memory access frequency and enables efficient hardware implementation.

#### BRAM-based weight storage and update

All synaptic weights were stored in BRAMs. During inference, weights were read from the BRAM and used to update the membrane potential. During training, the LReSuMe update values were written back to the same BRAM locations. This design enables on-chip training capability without external memory access and allows both inference and training to be performed within the FPGA architecture.

#### Multiplier-free datapath

The FPGA architecture was implemented using a multiplier-free datapath. Weight multiplication was replaced by shift-and-add operations to reduce hardware resource usage and power consumption. This design allows efficient implementation of both inference and training logic under fixed-point arithmetic.

#### Argmax classification

After temporal accumulation, output neuron responses were compared using an argmax operation. The neuron with the maximum response was selected as the predicted class label. This decision mechanism avoids softmax computation and simplifies hardware implementation.

#### FPGA implementation and simulation

The proposed architecture was implemented using synthesizable VHDL and evaluated using Vivado simulation and post-implementation reports. The FPGA device called Zynq UltraScale+ ZCU104 Evaluation Board was selected as the synthesis target in Vivado, and hardware resource usage, timing, and power estimates were obtained from implementation reports. All experiments were conducted using simulation-based evaluation. No physical FPGA board deployment was performed. The design provides on-chip training capability through BRAM-based weight updates.

#### Fixed-point representation

Fixed-point arithmetic was used for neuron dynamics, weight storage, and training updates. Different precision configurations (Q6.10 and Q6.26) were evaluated to analyze the impact of numerical precision on training stability and classification accuracy.

### Quantification and statistical analysis

Classification accuracy was used as the primary evaluation metric. Accuracy was computed as the ratio of correctly classified samples to the total number of evaluated images. Training dynamics were summarized using descriptive statistics, including mean values, standard deviation ranges, and maximum values, as shown in the corresponding figures. These statistics were computed directly from deterministic simulation outputs. For noise robustness evaluation, a randomly selected subset of the MNIST test dataset was used, as described in the main text. Noise was injected at predefined noise levels, and classification accuracy was computed for each condition. Hardware performance metrics, including resource utilization and power consumption, were obtained from Vivado post-implementation reports. Throughput was calculated based on simulation timing.
